# Supplementary figures and images for: Nomogram for predicting cancer specific survival in inflammatory breast carcinoma: a SEER population-based study
Source: PeerJ. 2019 Sep 16;7:e7659. doi: 10.7717/peerj.7659 (PMC6752187; doi:10.7717/peerj.7659)

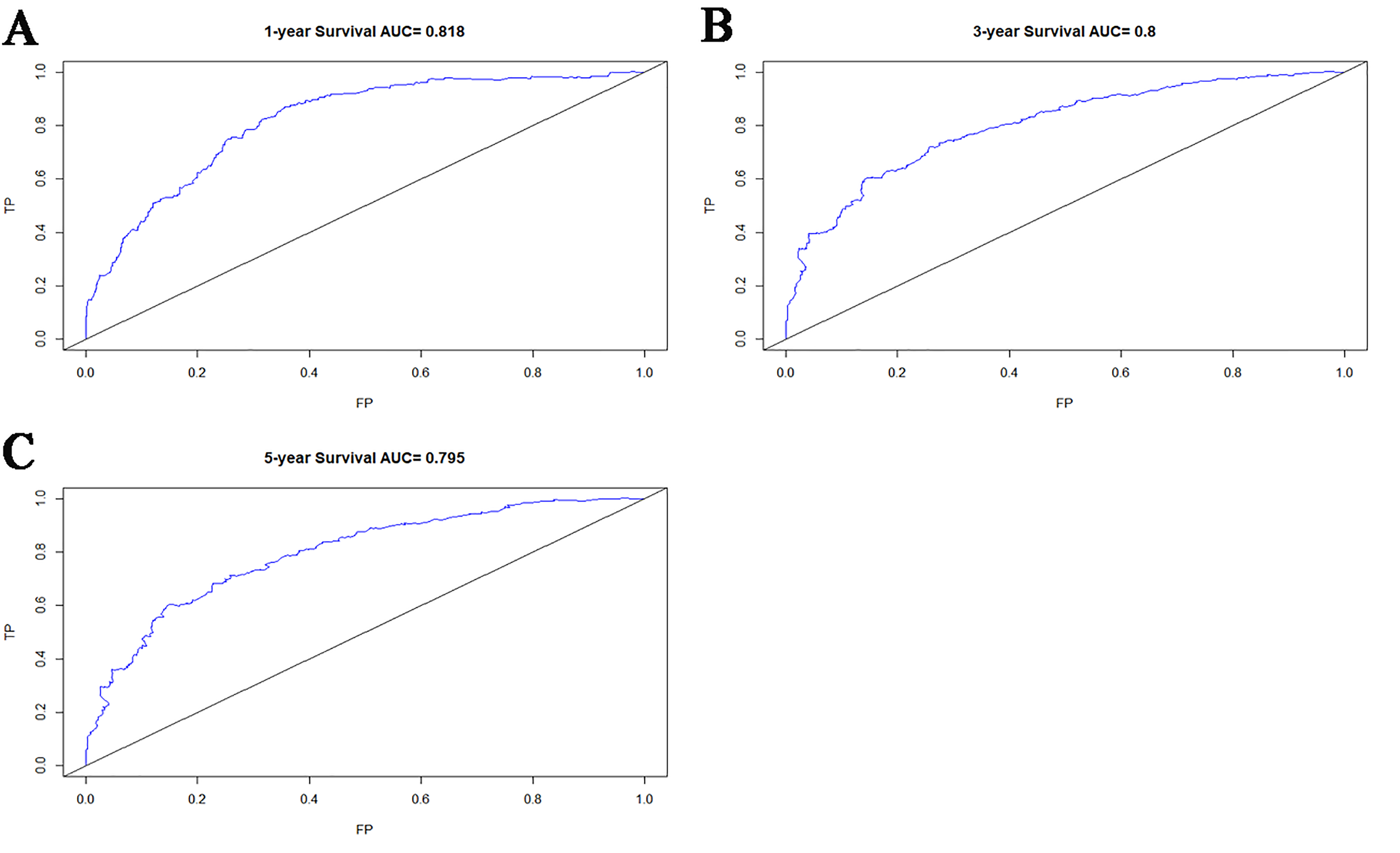

Supplement: Figure S1 — (A) 1-year, (B) 3-year, and (C) 5-year ROC curve [file peerj-07-7659-s001.png]

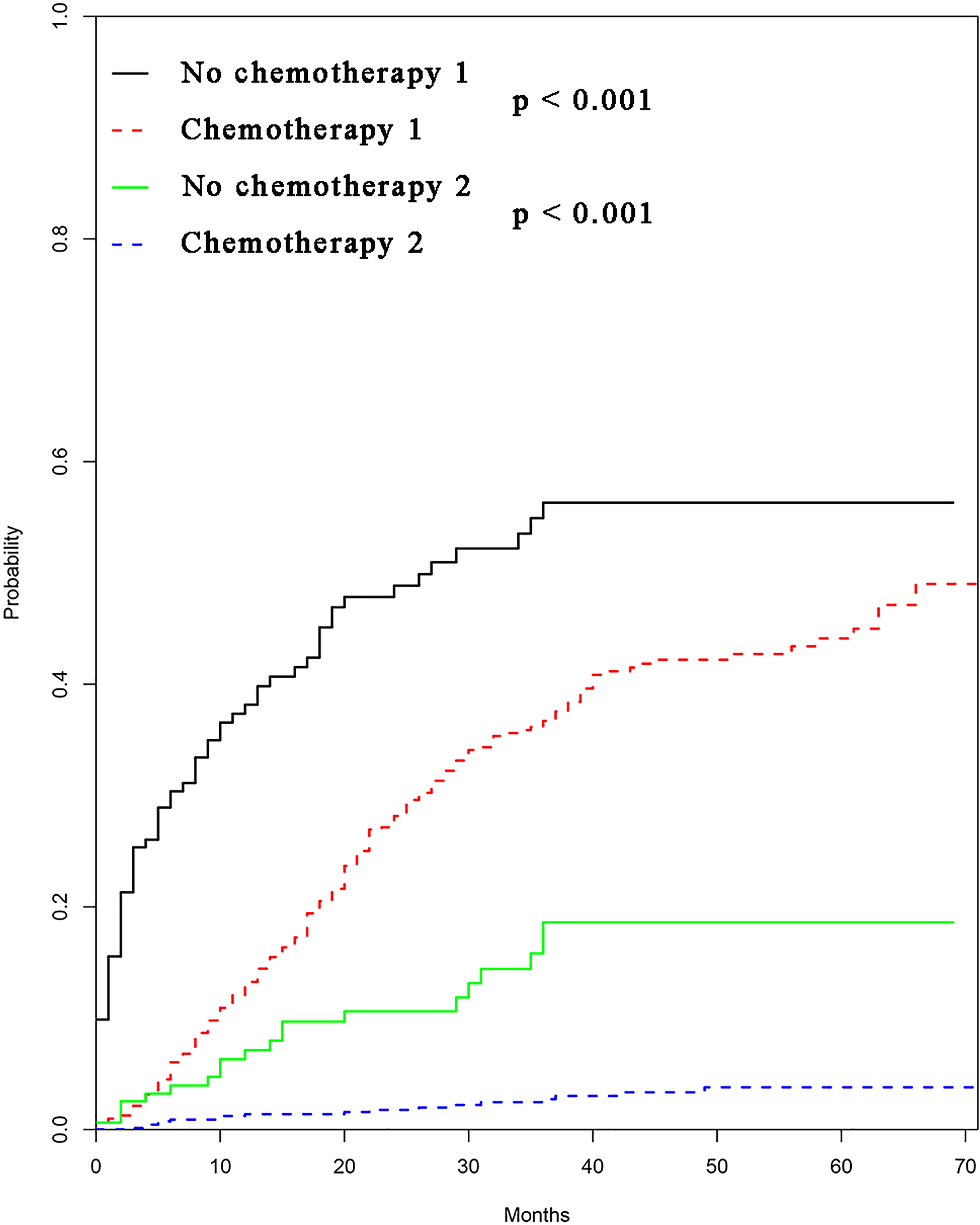

Supplement: Figure S2 [file peerj-07-7659-s002.png]

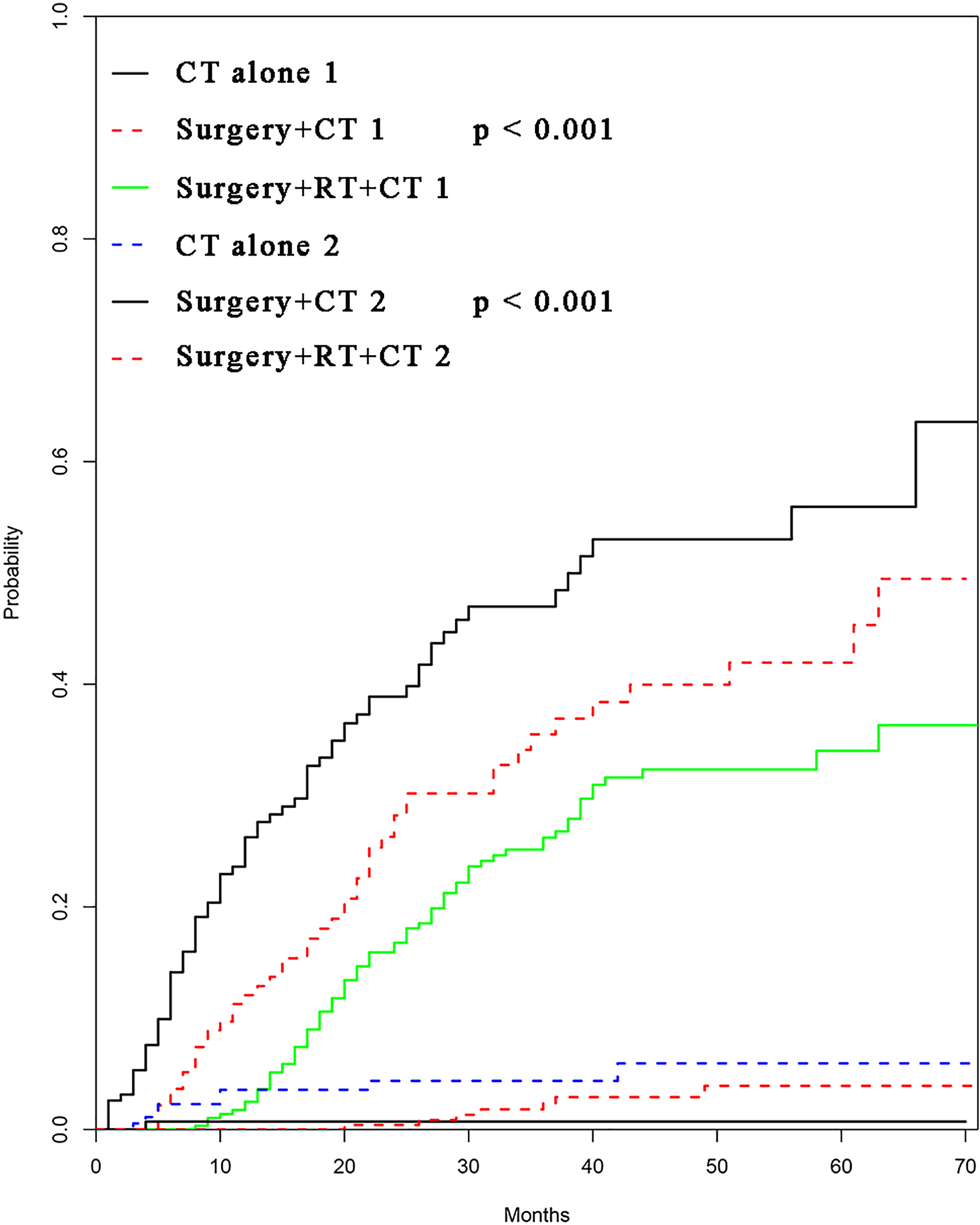

Supplement: Figure S3 [file peerj-07-7659-s003.png]
